# Adsorption Study of Congo Red Dye from Synthetic Wastewater at Different Concentrations Using Zinc Sulfide Nanoparticles

**DOI:** 10.3390/ma15145048

**Published:** 2022-07-20

**Authors:** Mohamed Rashad, Saloua Helali, Shams Issa, Saleh Al-Ghamdi, Marwah Alsharif, Ahmed Obaid Alzahrani, Mohamed Sobhi, Antoaneta Ene, Alaa M. Abd-Elnaiem

**Affiliations:** 1 Nanotechnology Research Laboratory, Department of Physics, Faculty of Science, University of Tabuk, Tabuk 71491, Saudi Arabia; s.helali@ut.edu.sa (S.H.); sh_issa@ut.edu.sa (S.I.); saalghamdi@ut.edu.sa (S.A.-G.); malsharif@ut.edu.sa (M.A.); 2 Physics Department, Faculty of Science, Assiut University, Assiut 71516, Egypt; abd-elnaiem@aun.edu.eg; 3 The Center of Energy Research and Technology (CRTEn), Hammam-Lif 2050, Tunisia; 4 Physics Department, Faculty of Science, Al-Azhar University, Assiut 71452, Egypt; 5 Center of Nanotechnology, King Abdulaziz University, Jeddah 22254, Saudi Arabia; profaaa052@gmail.com; 6 Physics Department, Faculty of Science, King Abdulaziz University, Jeddah 22254, Saudi Arabia; 7 Chemistry Department, Faculty of Science, Benha University, Benha 13511, Egypt; prof.m700@gmail.com; 8 Chemistry Department, Faculty of Science, University of Tabuk, Tabuk 71491, Saudi Arabia; 9 INPOLDE Research Center, Department of Chemistry, Physics and Environment, Faculty of Sciences and Environment, Dunarea de Jos University of Galati, 47 Domneasca Street, 800008 Galati, Romania

**Keywords:** ZnS, nanoparticles, structural parameters, adsorption, Congo red

## Abstract

Zinc sulfide (ZnS) nanoparticles were fabricated using the chemical precipitation method. The X-ray diffraction (XRD), Raman spectroscopy, and scanning electron microscopy (SEM) techniques were used to investigate the structural parameters of the formed ZnS. The hexagonal crystal structure of the Zn and ZnS phases was formed. The average crystallite size of the ZnS phase is 10.3 nm, which is much smaller than that of the Zn phase (54.5 nm). Several frequencies and phonon modes were detected in the Raman scattering spectrum belonging to the ZnS nanoparticles. The synthesized ZnS nanoparticles were used as catalysts to eliminate the Congo red (CR) dye, with different concentrations, from synthetic wastewater. The impact of the CR dye concentration and shaking period on the adsorption of CR was thoroughly investigated, and various adsorption kinetic models were tested. After 3 h of shaking, the adsorption efficiency reached 26.01% for 40 mg/L CR dye and 27.84% for 20 mg/L CR dye. The adsorption capacities of the CR dye in the presence of ZnS are 16% and 9% for 40 and 20 mg/L, respectively. Based on the correlation factor, the intraparticle diffusion kinetic model was considered the best of the tested models.

## 1. Introduction

Zinc sulfide (ZnS) is classified as an IIB–VIA semiconductor, and its electronic properties, like those of other materials, are determined by structural parameters. For example, ZnS typically crystallizes in hexagonal wurtzite or a cubic sphalerite structure, with a broad bandgap of 3.7–3.9 eV or 3.3–3.9 eV, respectively [1,2,3,4,5]. Furthermore, ZnS exhibits high exciton binding energy (0.04 eV), a small Bohr radius, and high activity [1]. As a result, the ZnS can be used as a catalyst, in UV–visible light-emitting diodes, flat-panel shows, infrared windows, etc. [1,2,3,4,5,6,7,8,9,10,11,12]. Because of the numerous applications for ZnS, significant effort has been spent on the preparation of ZnS nanostructures using various methods such as solid-state reaction, chemical precipitation, microwave-assisted solvothermal, hydrothermal, etc. [1,2,3,4,5,6,7,8,9]. The technique of synthesis has a considerable impact on the shape and control of the final product of ZnS, i.e., fabricating ZnS as bulk or nanostructures [1,2,3,4,5,6,7,8,9,10,11,12,13,14,15,16,17]. There is a large folder in the literature that studies nanostructured ZnS and its properties and applications. For example, Cheng et al. [4] prepared ZnS samples using hydrothermal methods and studied their structural parameters using experimental and theoretical studies on first- and second-order Raman analysis. The ultrasonic spray pyrolysis is utilized to produce ZnS thin films to be used as an n-type layer in low-cost Cu_2_SnS_3_/ZnS thin-film heterojunction solar cells [10]. Similarly, a more reactive and less expensive UV sensor based on a ZnS/p-Si heterojunction produced through chemical bath deposition has been described [11]. The investigation of optical phonon modes and their overtones in resonant Raman scattering by ZnS or ZnS and ZnO quantum dots produced using the Langmuir–Blodgett process has been presented [12,13]. Peng et al. successfully synthesized ZnS in various nanostructured forms such as tetrapods, nanorods, nanobelts, and nano-slices using the radio-frequency thermal plasma method in a wall-free manner [16]. The structural characteristics and morphology of ZnS nanostructures such as microparticles, nanoparticles, nanorods, and nanowires generated at low temperatures through a single-step hydrothermal technique were studied [17]. However, there are some materials still fighting against simple synthesis methods, low-cost raw materials, eco-friendly co-products, and large-scale production such as graphene [18] or graphite [19]. Moreover, the photochemical degradation of industrial dyes and antimicrobial activity could be done for significant results as antibiotics [20].

Growing urbanization and industrial growth have resulted in a huge number of contaminants, such as methylene blue (MB), methyl orange (MO), congo red (CR), and other dyes, being dumped into environmental water bodies. As a result, there is an ongoing need to find an efficient solution for removing such dyes for water purification. Purification procedures such as ozonization, chlorination, and filtering have their own set of constraints in terms of energy sources and toxic waste output. Another method of the water-purification process is the catalyst degradation, which employs a variety of materials such as semiconductors and nanocomposites. In the literature, there exist many catalysts based on diverse materials, including metal oxide semiconductors and nanocomposites, for the photodegradation and decolorization of CR from wastewater [21,22,23,24]. For example, after 4 h of sunlight irradiation, nanocomposites of reduced graphene oxide and CdS exhibit an excellent photocatalytic efficiency of 90% and a total decolorization ratio of 94.8%, demonstrating that wrapping CdS with reduced graphene oxide sheets can greatly improve its photocatalytic performance [21]. The degradation rate was reached 95.57% for the photodegradation of 100 mL of 30 mg/L CR for 1 h using 12 mg of Cu_2_O/-Fe_2_O_3_ catalyst [22]. Under simulated sunlight irradiation, the WO_3_-Cu hybrid demonstrated enhanced photocatalytic capacity for CR photodegradation [23]. Similarly, under UV light irradiation, the CR was decolorized to 35% in 2 h over 1% Ag/30% TiO_2_/SiO_2_ [23]. The low catalytic activity was explained by the highly strong CR adsorption (~60%) over titania catalysts.

Adsorption kinetics investigations, on the other hand, are curiously compared to photocatalysis and are sometimes more successful than photocatalysis. Adsorption is the best and most complete technology for removing azo dyes from water and industrial wastewaters because of its capacity to remove these dyes at any concentration, ease of design, and low cost [25]. As a result, a deeper study into the utilization of various forms of adsorbents to remove this harmful element from industrial wastewaters is required. ZnS and ZnS-based compounds are examples of materials used to remove poisonous dyes, among other uses. For example, the catalytic performance of various dyes was investigated using ZnS catalysts prepared by the chemical precipitation method [5]. Doping ZnS with other materials, such as Mn^2+^ and Ni, improves its properties, allowing it to be used in optical, catalytic, and biosensor applications [15]. The hydrothermal loading of MoS_2_ nanoflakes improves the catalytic activity of ZnS for removing rhodamine B (RhB) dye under visible light irradiation [26]. Furthermore, CuO loading to ZnS entrapped on carbon framework PVA/Chitosan for visible-light photocatalysis for tetracycline degeneracy and anti-bacterial applications were synthesized using co-precipitation and ultrasonic-assisted techniques [27]. The photocatalytic activity of the ZnS–CuO/PVA/Chitosan catalyst was increased to 94.7%, compared to 40% for ZnS and 60% for CuO catalysts. Based on the catalytic process, ZnS nanospheres immobilized on activated carbon were used to remove aquatic organic pollutants. Catalytic performance is well known to be highly reliant on catalyst structure, sizes, dimensions, doping, and surface morphologies. It is envisaged that ZnS nanostructures would have improved catalytic activity due to a considerable increase in surface area, which is often lacking in their bulk components.

In this work, the chemical precipitation reaction technique was used to prepare ZnS nanostructures. X-ray diffraction (XRD), scanning electron microscope (SEM), and Raman spectroscopy were used to investigate the structural parameters. At various CR dye concentrations, the prepared ZnS nanostructured was applied as a catalyst for removing CR from wastewater by shaking method. Various adsorption kinetic models, such as pseudo-first-order, pseudo-second-order, and intraparticle diffusion kinetic models, are extensively examined to explain and clarify the catalytic performance of ZnS nanoparticles. Furthermore, a concise comparison of the catalytic effectiveness in the present work with other publications related to ZnS prepared by various techniques and used as a catalyst for various dyes removal was presented.

## 2. Materials and Methods

### 2.1. Materials Preparation

The chemical precipitation method was used to prepare the ZnS nanoparticles. For this purpose, 1 M of ZnCl_2_ and 1 M of Na_2_S were dissolved in the distilled water. Then, according to the chemical equation Na_2_S (aq) + ZnCl_2_ (aq) → ZnS (s) + 2 NaCl (aq), the white ZnS samples were precipitated. The precipitates were washed in double-distilled water and separated by centrifugation. Then, they were dried at 100 °C for 5 h in a thermostat drier.

### 2.2. Materials Characterization

XRD was performed for ZnS powder using a Shimadzu (Kyoto, Japan) XD-3A X-ray diffractometer with monochromatic CuK_α_ radiation (λ = 1.5418 Å). For Raman spectroscopy, a stabilized DPSS-Laser with 532 nm was used for the excitation of ZnS powder. The morphology of ZnS powder was investigated using the SEM model SEM-JOEL. According to our previous research [28,29], the catalytic study was taken to the test in the following way: a solution of 1 L of CR (C_32_H_22_N_6_Na_2_O_6_S_2_) was prepared and then was diluted to prepare two concentrations of 20 mg/L and 40 g/L. A fixed quantity of ZnS nanoparticles of 2 mg was dispersed in 3.5 mL of the CR dye. The solution was moved to a shaking device model Lab Tech for various periods from 0 to 3 h. The flasks were shaken at 150 rpm until equilibrium was obtained. The CR dye absorbance, onto the ZnS that existed in the solution, was determined at room temperature using a Jenway 6300 UV–visible spectrophotometer (UK) in the wavelength range of 200–1100 nm.

### 2.3. Calculation of Catalytic Constants

It is well-known that the concentration of dye is directly proportional to the optical absorption values at a given wavelength, which is determined by the decrease in absorbance of dye after shaking for a given time. The percentage of adsorption efficiency of CR dye was determined as the ratio of change in absorbance (A_0_-A) to the initial absorbance (A_0_) [28,29,30]. The amount of CR dye per unit mass of ZnS nanoparticles ($q_{e}$ in mg/g) at equilibrium was computed utilizing: $q_{e}=\frac{C_{0}-C_{e}}{m}\;V,\;$where $C_{0}$ is the initial concentration of CR (mg/L), $C_{e}$ is the equilibrium concentration of CR (mg/L), *V* is the volume of the solution (L), and *m* is the mass of adsorbent used (mg). The same method was used for determining the amount of dye adsorbed at any time (*t*), $q_{t}$ (mg/L), and then analyzed using: $q_{t}=\frac{\left(C_{0}-C_{t}\right)}{m}V$, where $C_{t}$ is the concentration of CR (mg/L) at any time (*t*). The adsorption kinetics offers an idea of the adsorption mechanism, which is used to evaluate the process efficiency. The kinetics of experimental adsorption data of CR on ZnS was analyzed utilizing three models. The first model is the pseudo-first-order model, which is frequently used to estimate the kinetics and is given as follows [31,32,33,34]: $\frac{dq_{t}}{dt}=K_{1}\left(q_{e}-q_{t}\right)\overset{integration\;}{\to }\;\log\left(q_{e}-q_{t}\right)=log(q_{e})-K_{1}\frac{t}{2.303}$, where $K_{1}$ is the pseudo-first-order constant (min^−1^). The second model is the pseudo-second-order model, which is expressed as [35]: $\frac{dq_{t}}{dt}=K_{2}{\left(q_{e}-q_{t}\right)}^{2}\overset{integration\;}{\to }\;\frac{t}{q_{t}}=\frac{1}{K_{2}q_{e}{}^{2}}+\frac{t}{q_{e}}$, where $K_{2}$ is the pseudo-second-order constant (g/mg·min). The third model used in this work is the intra-particle diffusion kinetic, which is defined by the following equation [36,37]: $q_{t}=K_{diff}\sqrt{t}+C$, where $K_{diff}$ is the intraparticle diffusion kinetic model (mg/g·min^½^) and C is the kinetic parameter constant. The Elovic equation can also be used to study the adsorption of solutes from liquid solutions. The following formulation describes the Elovic equation [38,39]: $\frac{dq_{t}}{dt}=\alpha e^{-\beta q_{t}}\;\overset{integration\;}{\to }\;q_{t}=\frac{\ln\left(\alpha \beta \right)+\ln\left(t\right)}{\beta }$, where α is the initial sorption rate (mg/g·min), β is the desorption constant (g/mg). The α and β values are related to the surface coverage degree and activation energy for chemisorption. The adsorption process is commonly assumed to be divided into several steps, including (i) solution bulk transport, (ii) film diffusion, (iii) particle diffusion, and (iv) particle and solid surface sorption and desorption [28]. The diffusion mechanism during the adsorption process can be calculated using the Boyd equation [37,38]: $B_{t}=-0.4977-\ln\left(1-\frac{q_{t}}{q_{e}}\right)$.

## 3. Results and Discussions

### 3.1. Structure and Morphology

**Figure 1:**
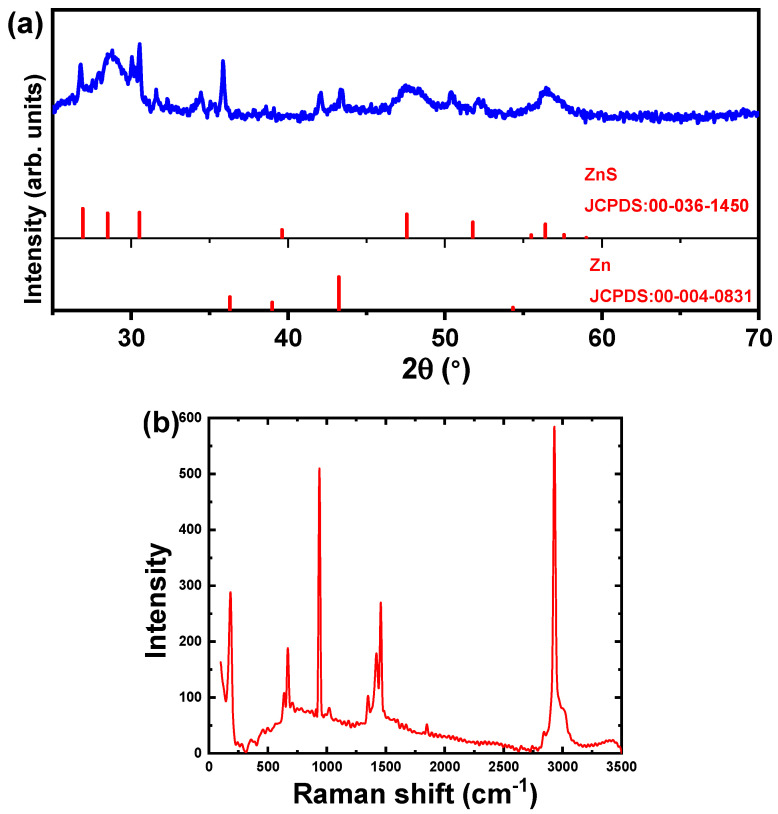
(**a**) XRD chart and (**b**) Raman spectrum of ZnS nanoparticles.

The XRD chart of the produced hexagonal ZnS nanoparticles along with the JCPDS cards for Zn and ZnS are shown in Figure 1a. The prepared ZnS is polycrystalline and consists of Zn and ZnS phases, which is consistent with most published works [6,26]. There are three observed diffraction peaks for the pure Zn phase, which are observed at 2θ of 36.3°, 39.0°, and 43.2°, which belong to the Miller indices of (001), (100), and (101), respectively. The detected diffraction peaks correspond to the hexagonal crystal structure, space group P63/mmc, and number 194 of the hexagonal crystal structure. The lattice constants a, b, and c are 2.665 Å, 2.665 Å, and 4.947 Å, respectively. The detected crystal structure is well agreed with the JCPDS card no. 00-004-0831. In addition, the wurtzite hexagonal ZnS phase is characterized by six diffraction peaks, which are located at 2θ of 28.5°, 30.5°, 39.6°, 47.5°, 51.77°, and 56.4°, respectively, and correspond to Miller indices of (002), (101), (102), (110), (103), and (112). The detected peaks correspond to a hexagonal crystal structure with the space group P63mc and the number 186. The lattice constants a, b, and c are 3.821 Å, 3.821 Å, and 6.2573 Å, respectively. The crystal structure matches with the JCPDS card no. 00-036-1450 which related to hexagonal ZnS. There are similar crystal structures of the ZnS samples formed by the thermal decomposition, hydrothermal, or co-precipitation methods that are available elsewhere [3,16,40]. Other structures, such as cubic crystal structure, were observed for the ZnS formed by the ball milling method [41,42].

Moreover, the structural parameters can be deduced from the XRD chart such as the average crystallite size (*D*) that can be determined using the Scherrer equation [43,44]: $D=\frac{0.9\lambda }{\beta_{hkl}cos\theta }$, where λ is the wavelength of radiation employed in Cu-K_α_ (1.5406 Å), $\beta_{hkl}$ is the full width at half-maximum (in rad), and θ is the angle at the maximum peak’s position (in rad). In addition, the micro-strain (ε) and the dislocation density (δ) can be evaluated from the modified Debye–Scherrer formula [44]: $\beta cos\theta =\frac{0.9\lambda }{D}+4\varepsilon sin\theta$, and $\delta =\frac{1}{D^{2}}$. For the investigated sample, the average crystallite size for Zn, and ZnS phases calculated using the Scherrer equation are equal to 54.5 nm, and 10.3 nm, respectively. The average micro-strain and dislocation density were estimated and found to be equal to 4.8 × 10^−4^, 3.37 × 10^−4^ nm^−2^, and 1.8 × 10^−3^, 9.4 × 10^−3^ nm^−2^ for Zn and ZnS phases, respectively.

Figure 1b depicts the Raman scattering spectrum for the chemical precipitated ZnS nanoparticles performed in air and at ambient temperature in the spectral range of 3500–100 cm^−1^. The Raman spectrum shows that several frequencies and phonon mode assignments can be detected. For example, at 354 cm^−1^, there is a visible band that could belong to the second-order longitudinal optical mode. The third-order longitudinal optical mode is represented by a band that occurs at 1018 cm^−1^ by a small peak. In addition, a small peak at 461 cm^−1^ has been detected, which is correlated to longitudinal optical and longitudinal acoustic waves. There are other lesser peaks recognized as pure acoustic or optical modes, as well as combinations of these, in addition to this longitudinal optical peak. Higher-order combinations of these fundamental modes make up the wideband centered at about 630 cm^−1^. It is worth noting that the measurement acquisition time under near-resonant scattering is two to three orders of magnitude faster than under green excitation conditions, on the order of seconds versus minutes. The macroscopic electric field related to the longitudinal optical vibrations in polar crystals, such as ZnS, causes the longitudinal optical mode energy to be larger than the transverse optical mode energy. This effectively eliminates the triple degeneration in the Brillouin zone center, resulting in double transverse optical mode degeneration and single longitudinal optical mode degeneration. Double degenerate transverse optical and nondegenerate longitudinal optical phonons are the optical modes we anticipate finding in first-order Raman scattering of a bulk sample. Calculations and experimental tests using polarized Raman or neutron scattering have determined the frequencies of these modes in ZnS [45,46,47]. At greater Raman shifts, there are additional peaks, the first of which being a group of three peaks approximately at 1400 cm^−1^. At 1750 cm^−1^, a minor peak was found, but at 2900 cm^−1^, a peak with a larger intensity was observed. These three observed peaks might correspond to longitudinal optical phonons of the third, fourth, and fifth orders, respectively [12,17].

**Figure 2:**
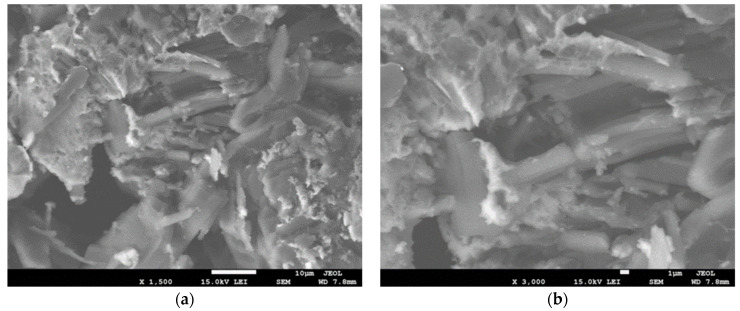
SEM images of the ZnS nanoparticles: (**a**) lower, and (**b**) higher magnifications.

Figure 2 displays SEM images of ZnS nanoparticles at different magnifications, 1500 and 3000 times, respectively, for Figure 2a,b. It is obvious from the SEM micrographs that the ZnS nanoparticles are distributed in a relatively regular manner. The surface morphology of ZnS samples is commonly seen to display two diverse morphologies such as rods, or belts, together with inhomogeneous shapes. Furthermore, two colors, e.g., bright and dark grey, may be detected, indicating the formation of two phases belonging to the Zn and ZnS phases, respectively, which confirms the XRD analysis results. The inhomogeneous, bright, and small size shapes are supposed to represent the Zn phase, whereas the rod-like particles are thought to represent the Zn phase. The average estimated agglomeration particle size for the Zn phase is 3.8 μm, while it is 2.5 μm for the size of the rod morphology or ZnS phase. The generated ZnS particles produced in this study may have morphologies that are like those produced by plasma and chemical vapor deposition methods, described elsewhere [16,44,47]. The developed shape in this work is comparable to the nanotubes of ZnS produced by the direct precipitation approach [48].

### 3.2. Catalytic Adsorption Studies

#### 3.2.1. Catalytic Adsorption of CR Dye

In recent years, a considerable number of researchers have focused on the synthesis of various categories of nanoparticles for catalytic application. As mentioned before, the distinctive properties of these nanoparticles including high adsorption qualify them in the category of competent catalysts. ZnS nanoparticles, in particular, with their broad bandgap and high excitation binding energy [5,15], are promising materials for the adsorption of organic dye pollutants.

**Figure 3:**
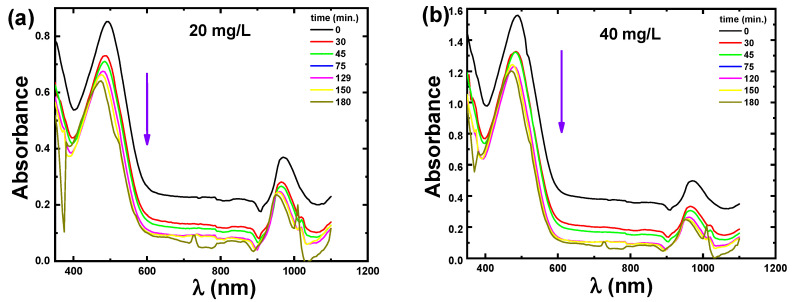
UV–visible absorption spectra of the ZnS nanoparticles for (**a**) 20 mg/L CR and (**b**) 40 mg/L CR dye at different shaking times. The inserted arrow shows the direction of the increase of shaking time.

Figure 3a,b display the absorption spectra of adsorption of 20 mg/L and 40 mg/L CR solutions using 2 mg ZnS nanoparticles as catalysts during various times of shaking. The diminution of CR absorption peaks at λ_max_ = 498 nm with the increasing of the shaking time reflects the dye adsorption. This diminution represents the potential for each stimulated substance to degrade. In addition, the decrease in the absorption of the CR solution resulted in the breaking of the homo and hetero-polyaromatic rings existing in the dye molecules. The influence of ZnS nanoparticles on the percentage adsorption of the CR dye has been investigated by altering the shaking time interval up to 3 h.

**Figure 4:**
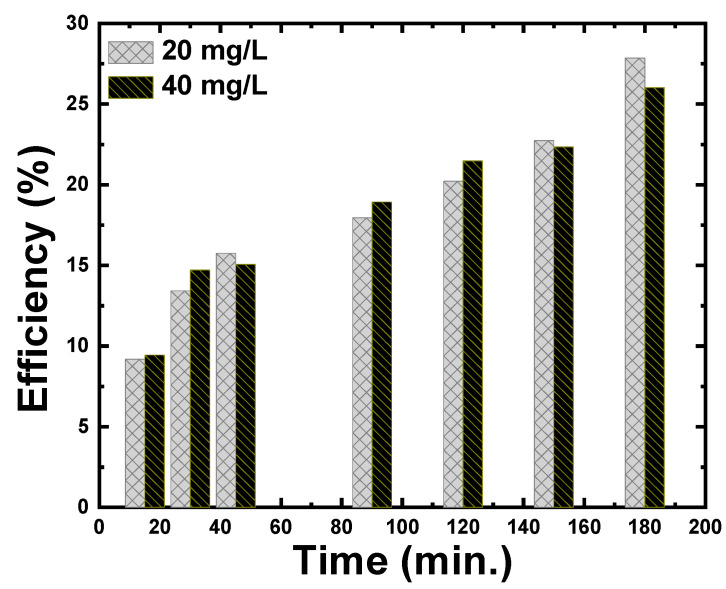
The catalytic efficiency at various shaking times of 20 mg/L and 40 mg/L CR dye using ZnS nanoparticles.

Figure 4 shows the plots of the percentage adsorption versus time for two concentrations of 20 mg/L and 40 mg/L of CR. After 3 h of the shaking, up to 27% of CR dye was degraded in the presence of 2 mg of ZnS nanoparticles. Furthermore, the figure demonstrates that the adsorption increases with increasing the dye concentration from 20 mg/L to 40 mg/L, especially for lower shaking times. This could be concluded as the amount of dye used increases, more dye molecules will be deposited on the surface of the catalysts, hence reducing the active sites of the catalysts. As a result, the formation of hydroxyl radicals will be reduced as the occupied space of the catalyst surface increases. Surprisingly, as the shaking time was increased above 150 min, the efficiency of adsorption of lower concentrations of the CR dyes improved.

#### 3.2.2. Comparison of the Catalytic Performance Using ZnS Nanoparticles

In this section, a comparison of the catalytic degradation of several dyes in the presence of the ZnS nanoparticles with existing works is illustrated. During the last decade, ZnS nanoparticles have attracted much attention due to their remarkable properties, such as direct and wide bandgap (3.7 eV) [15], two crystalline forms (Blende at room temperature, and Wurtzite at higher temperature), low toxicity, thermal stability. The ZnS nanostructures provide a diverse number of applications such as solar cells [10], biosensors, chemical sensors [11], and photocatalysis [27]. To further verify the potential application of ZnS nanoparticles as photocatalysts for studies of efficiently degrading dyes in aqueous industrial effluents [5,15], Table 1 compares and summarizes the results related to the variation in degradation efficiency using ZnS for various dyes. One can see that ZnS has the ultimate degradation of MB dye (92%) in 198 min. utilizing pure ZnS catalysts, for dye concentration of 12 mg/L [9]. However, at a time limit of UV irradiation of 180 min, only 72.13% degradation was found for pure ZnS catalysts produced via solid-state reaction, for a dye concentration of 10 mg/L [15].

After 14 h of UV irradiation, the ZnS showed an outstanding efficiency removal rate of 97.67% towards the MO when compared to commercial ZnS [2]. On the other hand, 26.61% of MO [2] can be degraded by the catalysis of commercial ZnS for dye concentration of 20 mg/L, as shown in Table 1. Under 2 h of UV irradiation, the ZnS generated by chemical precipitation techniques has removal effectiveness of 78.41, 81.22, 90.90, and 95.10% for eliminating MB, XO, MO, and MR dyes, respectively [5]. The starting ingredients in the microwave-assisted solvothermal method were critical in influencing the degradation efficiency of RhB dye [6,7]. The hydrothermal ZnS has high effectiveness towards RhB (90%) [8], and MB (92%) [9].

For example, in the adsorption approach for removing hazardous dyes, ZnS nanotubes were employed to remove CR, Aniline blue (AB), and Brilliant blue (G250), as well as MB, and Azophloxine (AR1) [46]. It was reported that adsorption is dependent on the dyes studied, and the greatest efficiency was 96.3% for the CR dye. In the present investigation, the concentration of CR dye has a minor effect on efficiency, however, the shaking period has a considerable effect on efficiency. The lower efficiency found in this study compared to others might be attributable to the different dyes, interactions between the adsorbent and dyes, surface area, and particle size as well as the amount of catalyst used.

#### 3.2.3. Effect of the Contact Time and the Initial Concentration on Adsorption of CR Dye

**Figure 5:**
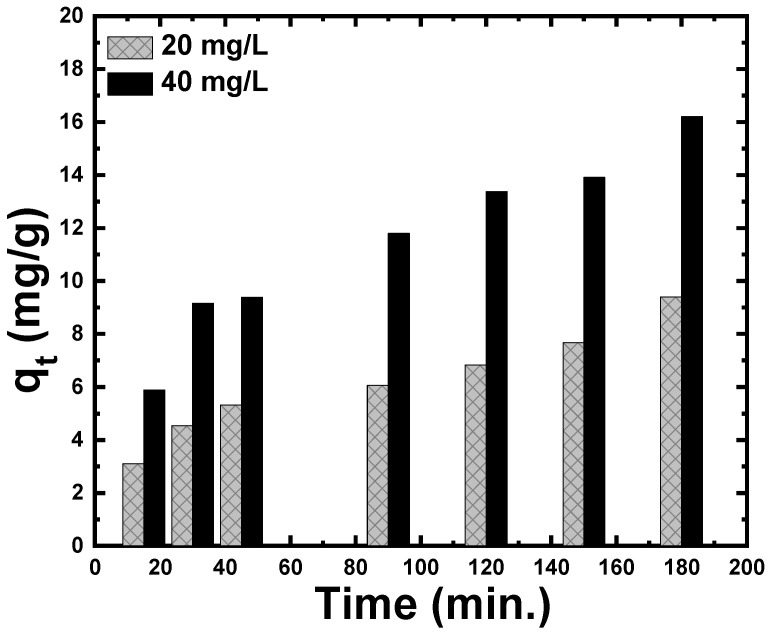
Adsorption capacity ($q_{t}$) of CR dye on the ZnS nanoparticles at different shaking times and different initial concentrations.

The adsorbed masses of CR per unit mass of ZnS nanoparticles at time t were calculated using the relation: $q_{t}=\frac{\left(C_{0}-C_{t}\right)}{m}V$. Figure 5 shows the influence of contact time and initial concentrations (20 mg/L and 40 mg/L) on CR adsorption. An increase in contact time increases the amount of CR adsorbed onto the surface of ZnS nanoparticles. Moreover, the behavior that defines the relationship between adsorption ($q_{t}$) and contact time is very close to the behavior that describes the relationship between catalytic efficiency (η) and time, being similar to the results of previous research. Furthermore, the initial concentration of CR dye has a significant impact on the value of $q_{t}$. The value of $q_{t}$ observed for a higher concentration of CR dye is much higher than that recorded for a lower concentration of CR dye.

This finding indicates that, in addition to adsorption, the dye adsorption on the catalyst plays a major role in water purification.

#### 3.2.4. Adsorption Kinetic Models

Adsorption kinetics is a fundamental aspect of analyzing the adsorption mechanism. The kinetics of adsorption of CR dye on ZnS nanoparticles was studied using the pseudo-first-order kinetic model, and pseudo-second-order kinetic model, as well as an intraparticle diffusion model. The computed parameters of the three kinetic models are tabulated in Table 2.

**Figure 6:**
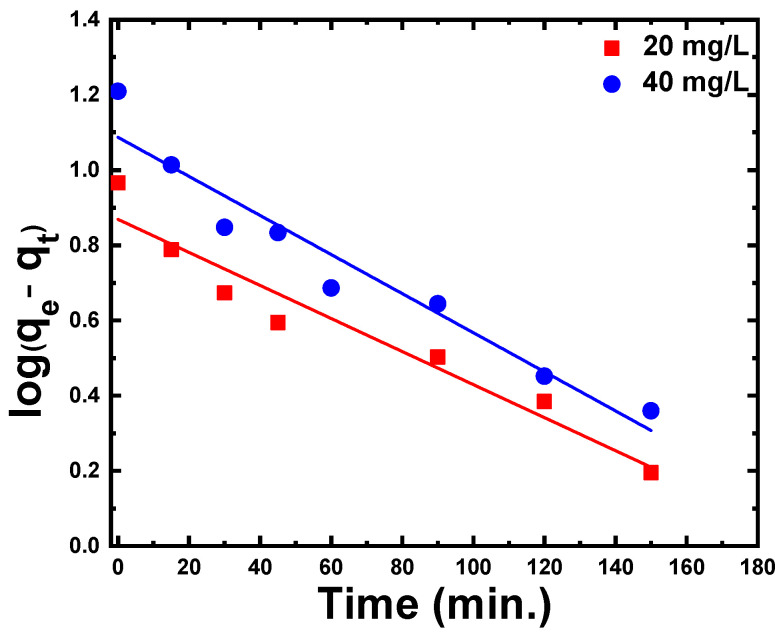
Pseudo-first-order kinetic model (log($q_{e}-q_{t}$) versus time) for CR dye adsorption on the ZnS nanoparticles.

The plot of log($q_{e}-$–$q_{t}$) versus time for various CR concentrations is shown in Figure 6 to evaluate the pseudo-first-order kinetics. The constants of pseudo-first-order model such as $K_{1}$ and $q_{e}$ were determined from the slope and intercept of the fitted lines, respectively. The correlation coefficient (R^2^) for the plot is equal to 0.94 for the two concentrations of CR but the calculated $q_{e}$ values from first-order kinetic were lower than the experimental $q_{e}$ value determined using the relation: $q_{e}=\frac{C_{0}-C_{e}}{m}\;V$. This demonstrates that the pseudo-first-order model was inadequate to estimate the adsorption kinetics of CR on ZnS nanoparticles.

**Figure 7:**
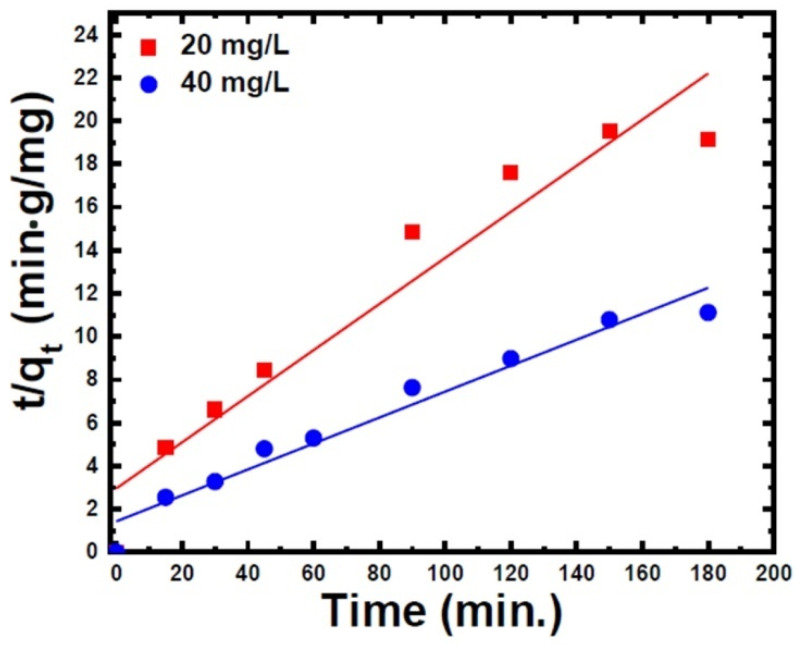
Pseudo-second-order kinetic model (t/$q_{t}$ versus time) for CR dye adsorption on the ZnS nanoparticles.

The pseudo-second-order model constants such as *K_2_* and $q_{e}$ could be determined by plotting *t*/$q_{t}$ versus time (Figure 7). Based on the linear fitting of plot t/$q_{t}$ versus *t*, the values of $K_{2}$ and $q_{e}$ were calculated from the slope and intercept, respectively. As shown in Table 2, the regression coefficient reached 0.96 for 40 mg/L of CR concentration, and the estimated $q_{e}$ values were close to their experimental values. These studies demonstrate that the CR adsorption on ZnS nanoparticles follows a pseudo-second-order kinetic model, indicating that the adsorption process was controlled by chemisorption. Similar results were also obtained by Zhang et al. [31].

**Figure 8:**
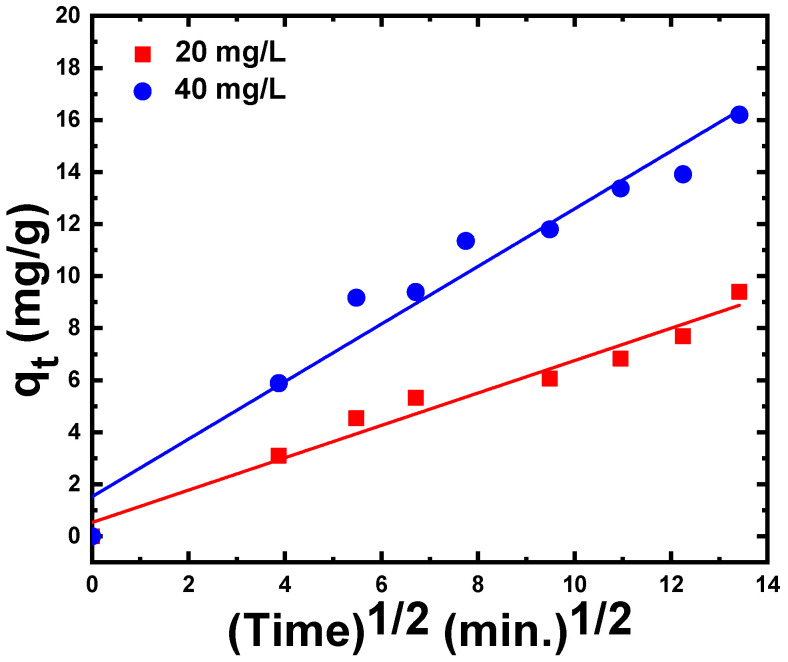
Intra-particle diffusion model ($q_{t}$ versus time^½^) for CR dye adsorption on ZnS nanoparticles.

The third model, intra-particle diffusion, is illustrated in Figure 8. As shown, the intra-particle diffusion result is fitted by a linear plot with a regression coefficient equal to 0.97 and 0.96 for 20 mg/L and 40 mg/L of CR dye, respectively. The corresponding values of the diffusion rate constant, $K_{diff.}$, and C are summarized in Table 2. This linear plot demonstrates that the adsorption of CR on ZnS nanoparticles has been directed by a single step. Furthermore, the diffusion curves did not pass through the origin of the coordinates, indicating that the surface adsorption and intra-particle diffusion played a role in the adsorption mechanism.

**Figure 9:**
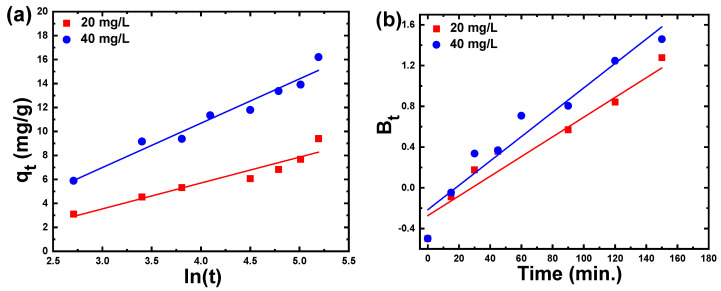
(**a**) Elovich kinetic model ($q_{t}\;$versus ln(t)) and (**b**) Boyd model ($B_{t}$ versus time) for adsorption of CR dye on ZnS nanoparticles.

The Elovich equation is extensively utilized in the sorption studies of dyes in aqueous solutions. The Elovich equation is frequently associated with the linear representation based on the plot of $q_{t}$ as a function of ln(t) (Figure 9a). From the slope and intercept of fitting lines, the magnitude of the sorption (α) and desorption (β) constants were found to be 60.24 mg/g.min; 0.065 g/mg and 80.28 mg/g.min; 0.038 g/mg for 20 mg/L and 40 mg/L of CR dye, respectively, as noted in Table 2. As the dual nature of the intraparticle propagation plot mentions the participation of both surface sorption and intraparticle diffusion, the results of the experiments were analyzed using the Boyd kinetic equation. As a result of the Boyd function, the estimated B_t_ values have been graphed against time as presented in Figure 9b. Adsorption rates are controlled by either external mass transfer or intraparticle diffusion, and the linearity of this figure may indicate which is the rate-controlling process. As demonstrated in Figure 9b, the straight line did not pass through the origin point, which allowed us to see that external mass transport was the main rate-controlling parameter of CR adsorption onto ZnS nanoparticles.

## 4. Conclusions

ZnS nanoparticles were synthesized using the chemical precipitation technique. The structural parameters of the formed ZnS were investigated using XRD, Raman spectroscopy, and SEM techniques. The presence of both hexagonal crystal structures of the Zn and ZnS phases confirms the polycrystalline ZnS nature. The average crystallite size of the ZnS phase equals 10.3 nm, which is much smaller than the 54.5 nm for the Zn phase. The average particle size in the Zn phase is 3.8 μm, while in the ZnS phase, it is 2.5 μm. The Raman scattering spectrum of ZnS nanoparticles shows a variety of frequencies and phonon modes. The synthesized ZnS nanoparticles were used as catalysts to remove the Congo red (CR) dye from wastewater. The effect of dye concentration and shaking time on CR adsorption is thoroughly investigated, and several models were tested. After 3 h of shaking time, the adsorption efficiency for 40 mg/L CR was 26.01% and 27.84% for 20 mg/L CR. Moreover, in the presence of ZnS, the adsorption capacities of the CR dye were 16% and 9% for 40 and 20 mg/L, respectively. The intraparticle diffusion kinetic model is the best of the tested models based on the correction factor. It was observed that adsorption processes contribute to the elimination of CR and hence the purification of water. The present work was compared to previous research on the use of ZnS nanoparticles as a catalyst for the removal of various dyes. The comparison of poisonous dye adsorption utilizing ZnS synthesized by various methods reveals the production process, starting mass of catalyst, dye removal, and initial concentrations of dye.

## Figures and Tables

**Table 1:** The catalytic efficiency of CR dye using ZnS nanoparticles in this study was compared to previously published works on other dyes.

Preparation Method	Dye (Symbol)	Dye Concentration (mg/L)	Catalytic Efficiency (%)	Catalyst Amount (g/L)	Time (min)	Ref.
Solid state reaction	Methylene blue (MB)	10	72.13	0.3	180	[15]
Salt-alkali-composited-mediated	Methyl orange (MO)	20	97.67 for synthesized ZnS& 26.61 for commercial ZnS	0.2	840	[2]
Chemical precipitation	MB, xylenol orange (XO), Methyl orange (MO), and methyl red (MR)	50	78.41, 81.22, 90.90 & 95.10	0.5	120	[5]
Microwave-assisted solvothermal	Rhodamine B (RhB)	10	97, 82, and 56 prepared ZnS from zinc nitrate, zinc chloride, and zinc acetate	0.75	210	[6]
Chemical precipitation	Bromophenol blue (BPB)	10	42.5	2.5	180	[7]
Hydrothermal	Rhodamine B (RhB)	5	90	1.0	60	[8]
Hydrothermal	Methylene blue (MB)	12	92	0.1	198	[9]
Direct precipitation	Congo red (CR)	25	96.3	0.2	60	[48]
	Aniline blue (AB)		87.2			
	Brilliant blue (G250)		61.6			
	Methylene blue (MB)		83.4			
	Azophloxine (AR1)		6.5			
Chemical precipitation	Congo red (CR)	20	27.84	0.6	180	This Paper
		40	26.01			

**Table 2:** CR dye adsorption parameters on ZnS nanoparticles using various kinetic models (exp. = experimental; cal. = calculated).

Model	Parameter	Dye Concentration (mg/L)	
		20	40
**Pseudo-first-order kinetic model**	$q_{e}$ exp. (mg/g)	9.24	16.19
	$q_{e}$ cal. (mg/g)	7.38	12.19
	$q_{e}$ exp.– $q_{e}$ cal.	1.86	4
	$K_{1}$ (min^−1^)	0.01	0.012
	R^2^	0.96	0.97
**Pseudo-second-order kinetic model**	$q_{e}$ cal. (mg/g)	9.43	16.67
	$q_{e}$ exp.– $q_{e}$ cal.	0.19	0.48
	$K_{2}$ (g/mg·min)	0.0038	0.00252
	R^2^	0.96	0.97
**Intra-particle diffusion kinetic model**	$K_{diff.}$ (mg/min^1/2^·g)	0.62	1.106
	*C*	0.53	1.52
	R^2^	0.98	0.96
**Elovic kinetic model**	α (mg/g·min)	8.43	11.24
	*β* (g/mg)	0.46	0.27

## Data Availability

All relevant data are within the paper. The data presented in this study are available on request from the corresponding authors.
